# Duplicated Inferior Vena Cava in a Patient With Ampullary Adenocarcinoma: A Case Report and Literature Review of Anatomical Variations

**DOI:** 10.7759/cureus.11576

**Published:** 2020-11-19

**Authors:** King Tung Cheung, Enoch Wong

**Affiliations:** 1 Surgery, Eastern Health, Melbourne, AUS; 2 Surgery, Monash University Eastern Health Clinical school, Melbourne, AUS; 3 Surgery, Monash Univeristy Eastern Health Clinical School, Melbourne, AUS

**Keywords:** duplicated ivc, ampullary adenocarcinoma, whipple procedure

## Abstract

Duplicated inferior vena cava (IVC) is a rare anatomical anomaly as a result of failed regression of the left supracardinal vein during the embryonic stage. This anatomical variation has certain surgical implications and could lead to potential catastrophe perioperatively. We hereby report a case of a 54 years old male in whom a whipple procedure was performed with type 1 duplicated IVC for ampullary adenocarcinoma. Review of current literature of such anatomical anomaly will also be discussed. This venous anomaly must be kept in mind in all surgical procedures involving the retroperitoneum to minimise the risk of incomplete lymph node dissection and life-threatening bleeding, and to guide management for deep vein thrombosis in the post-operative setting.

## Introduction

Duplicated inferior vena cava (IVC) is a rare anatomical anomaly, with an overall incidence ranging from 0.2 to 3% [1, 2]. It is thought to be due to a persistent left supracardinal vein during the embryonic stage [3]. Certain anatomical variations have been reported. Patients with a duplicated IVC have their left and right iliac veins drained through the corresponding ipsilateral vena cava. These vena cava ascend along each side of the aorta and merge into one confluent IVC at the level of renal veins via the preaortic trunk. The resulting IVC then resumes the usual position from this point onward. Three main types of duplicated IVC exist. We report a case in which a whipple procedure was performed on a 54 years old male with type 1 duplicated IVC for ampullary adenocarcinoma. The potential surgical implications of such anatomical anomaly will also be discussed.

## Case presentation

**Figure 1:**
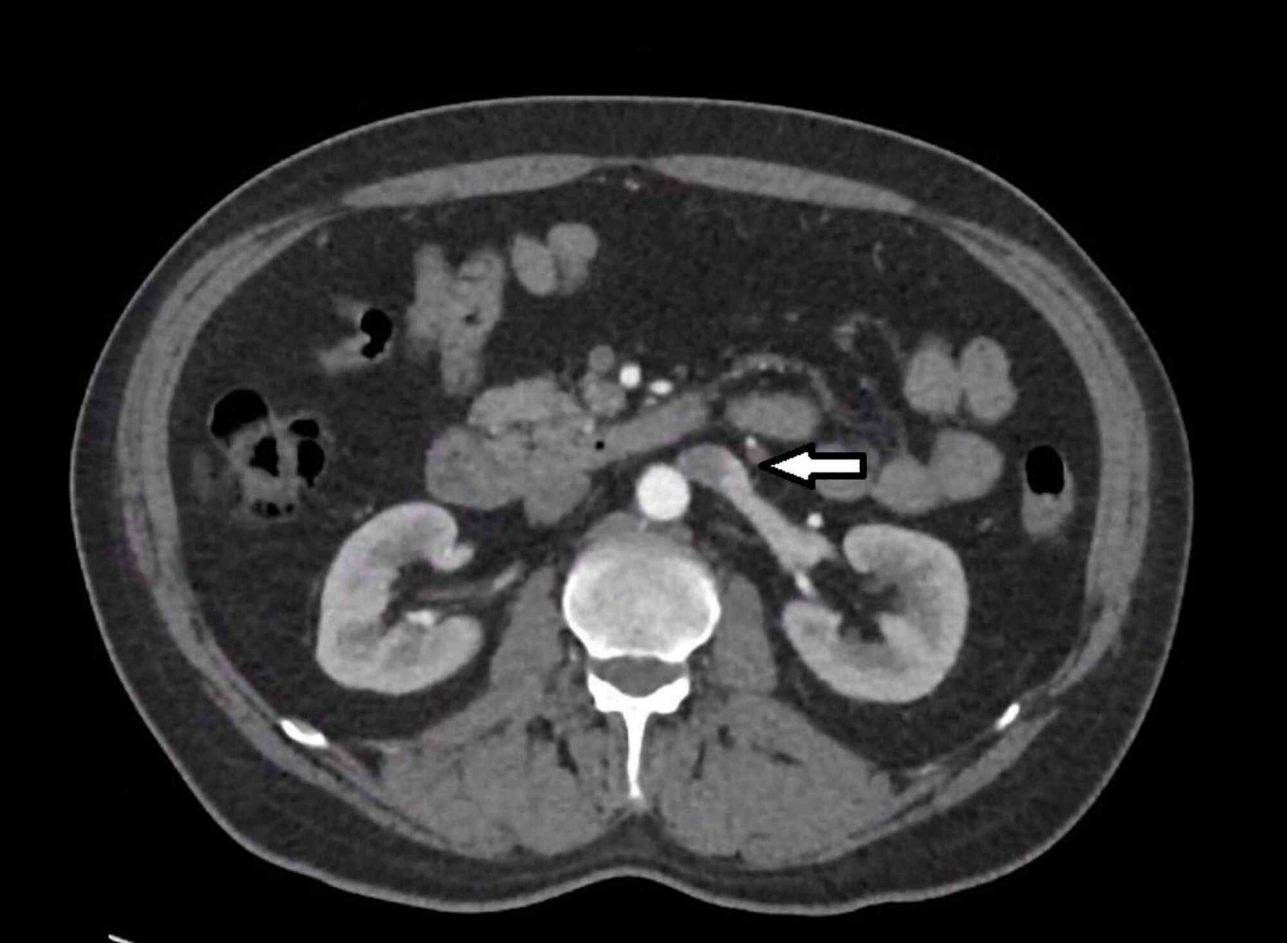
CT abdomen (axial): Bilateral vena cava on each side of the abdomen

**Figure 2:**
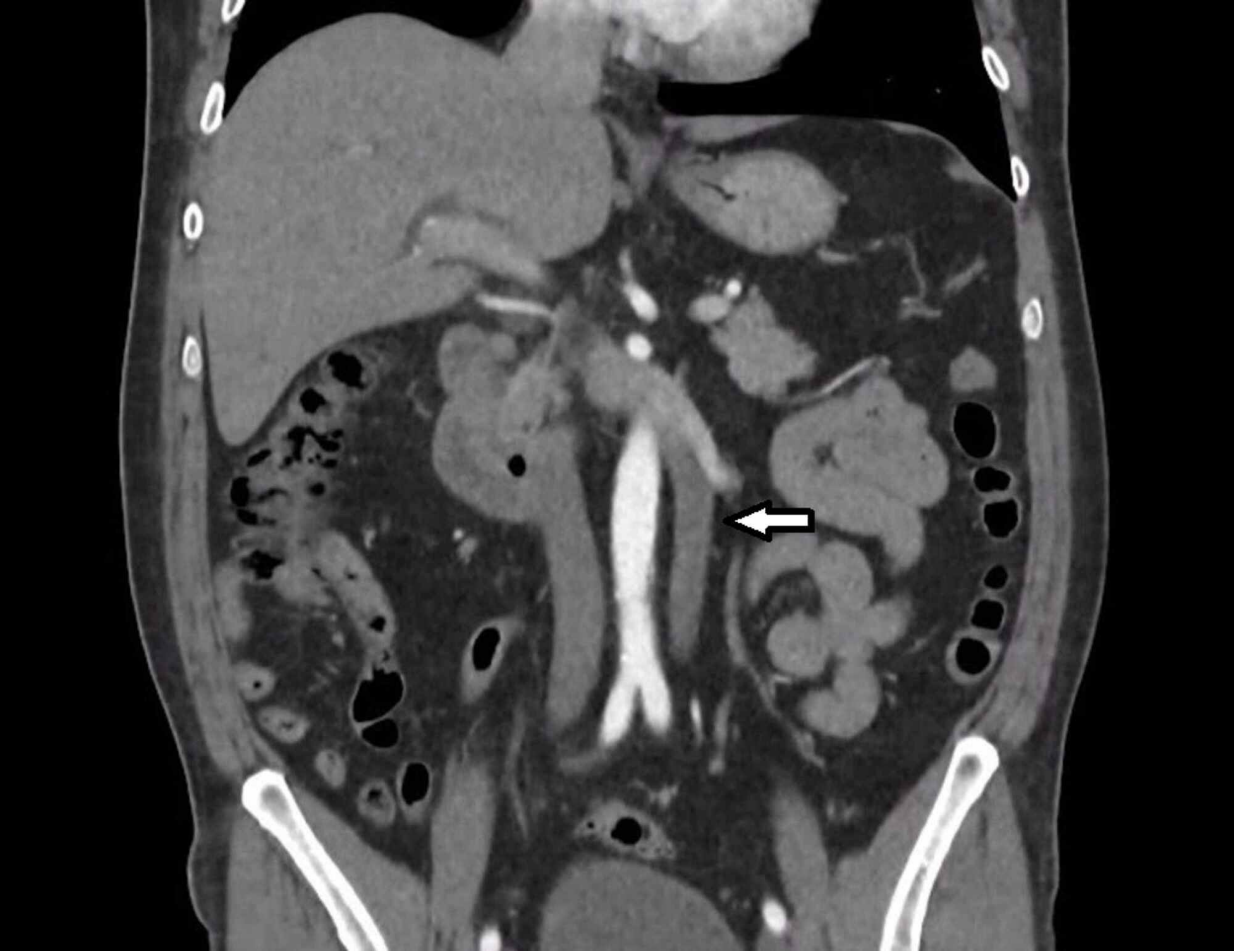
CT abdomen (coronal): Bilateral vena cava merging into the preaortic trunk.

We present a case of a 54 years old male who initially presented with one month history of worsening epigastric pain and vomiting. He was referred to our care after an ultrasound and CT abdomen showing dilated common bile duct up to 13mm with no obvious intraductal obstruction. An incidental type 1 duplicated IVC was demonstrated on the CT abdomen (Figure 1 and 2).

No intra-abdominal mass or lymphadenopathy were detected. Notably, he is a known BRCA2 mutation carrier. Both, his mother and sister had BRCA2-positive breast cancer. One of his great uncles also had male breast cancer. The patient was clinically jaundiced on presentation, with a bilirubin of 106. His other liver function tests were ALT at 465, GGT at 1032 and at ALP 271. His tumour markers CA-125 and CA-199 were 12 and 94 respectively.

**Figure 3:**
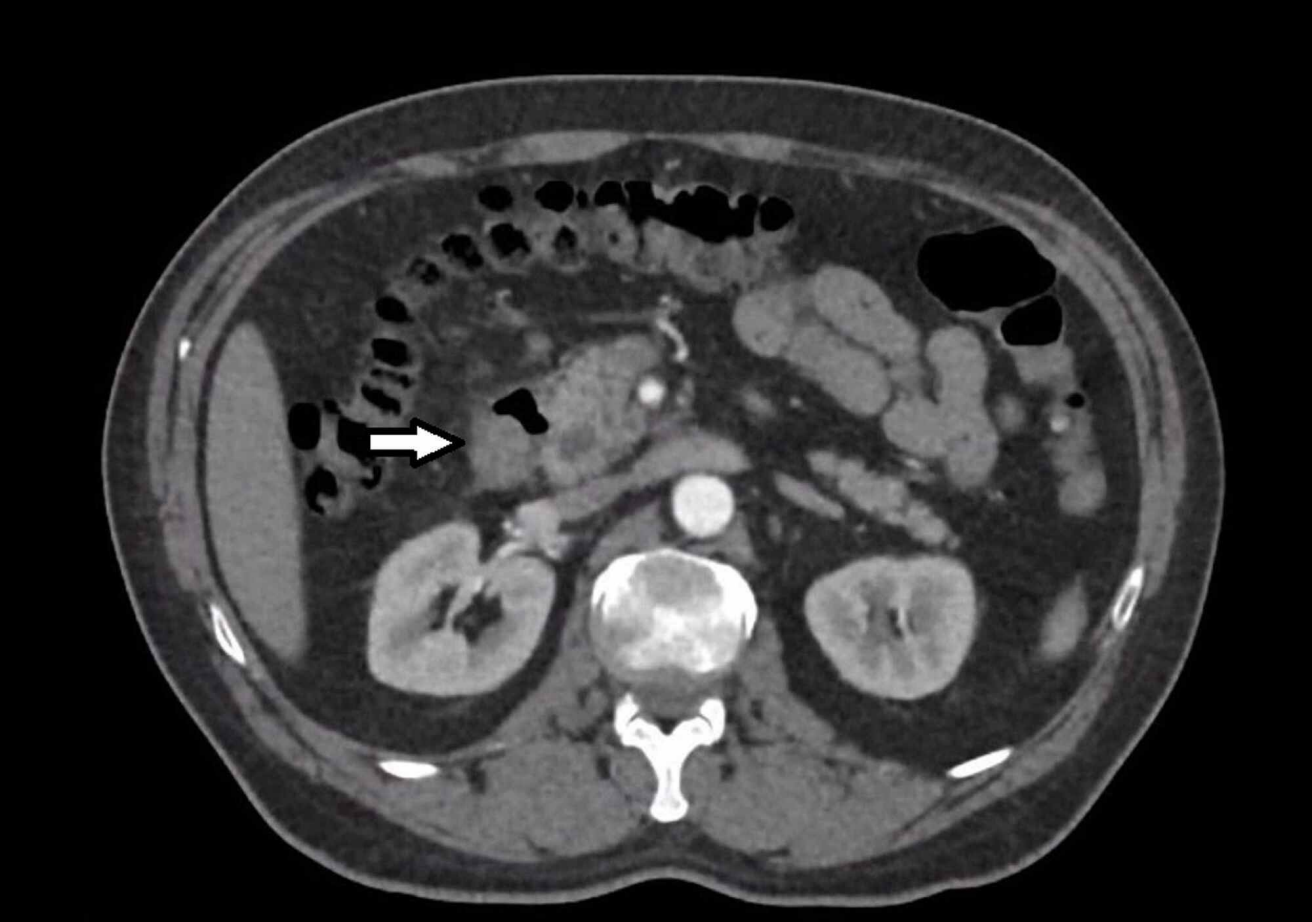
CT abdomen (axial): Bilateral vena cava merging into the preaortic trunk. Note in proximity an apparent cavitation, with air fluid level, at the medial aspect of the second part of duodenum.

The patient was subsequently found to be hypotensive and tachycardic with a new drop in haemoglobin from 100 to 72 during his stay. An urgent gastroscopy then revealed a 10mm punched-out ulcer with adherent clot at the ampulla. The lesion was partially obstructed and no active bleeding was seen. Histology on biopsy showed moderately differentiated adenocarcinoma. CT angiography was then performed. No active extravasation was seen and the gastroduodenal artery was empirically embolised with microcoils. An apparent cavitation at the medial aspect of the second part of duodenum was now identified on the CT imaging (Figure 3). No metastasis was seen on staging CT imaging.

A whipple procedure was performed. The duplicated right IVC was visualised intraoperatively with mobilisation of the uncinate process but appeared relatively normal. The left sided IVC was not encountered. No major complications were reported during his inpatient stay. Patient was discharged home and subsequently commenced on adjuvant therapy.

## Discussion

Duplicated IVC is a rare anatomical anomaly. Caval development commences in the 6th week of embryonic development. All three pairs of precursor venous systems, namely the posterior cardinal, the subcardinal and the supracardinal veins are formed by the end of the 8th week [3]. The renal segment of the IVC is formed by the fusion of the right suprasubcardinal and postsubcardinal veins. Duplicated IVC is most commonly thought to be the result of the persistence of both supracardinal veins, which constitute the infrarenal segment of the IVC. Three main variations exist, each with different calibres of the IVC trunks in relation to the preaortic trunk. Type 1 duplication is the most common form, in which both the bilateral vena cava, as well as the preaortic trunk, are of the same calibre. In type 2 duplication, the two symmetrical IVCs are smaller in calibre compared to the preaortic trunk. Type 3 duplication described a right IVC prominence, with the preaortic trunk being the largest of the three. Duplication of IVC, though rare, is important to be recognised perioperatively, especially surgical procedures involving the retroperitoneum [4-8].

**Figure 4:**
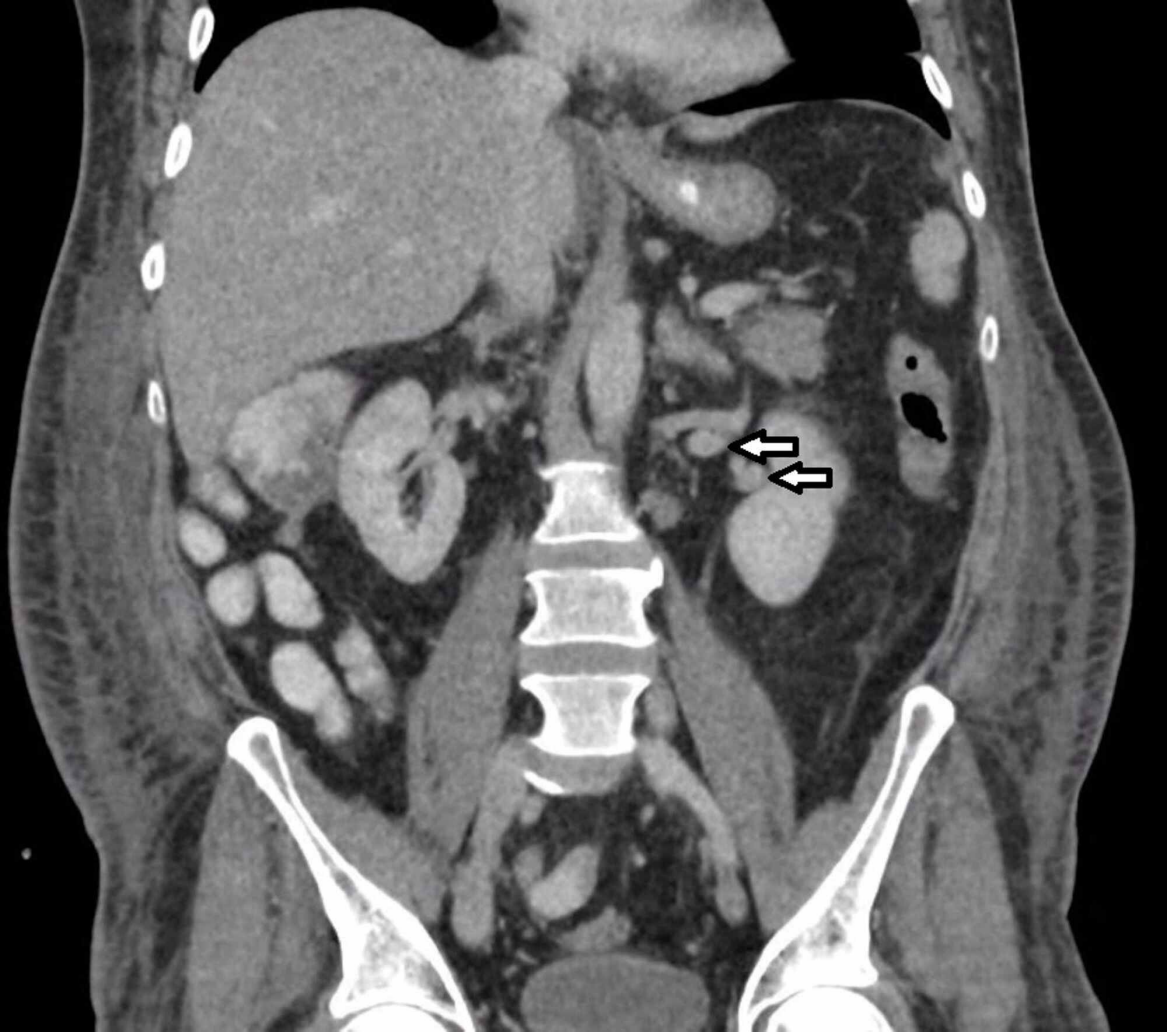
CT abdomen (coronal): Superior and inferior renal veins leaving the left kidney.

The genitourinary vasculature and anatomy vary in patients with duplicated IVC. Right renal veins are often multiple. The most common anomalies on the left side include retroaortic and circumaortic left renal vein, each with different specific subtypes [9]. Horseshoe kidney may occasionally be observed. Our patient has a normal renal vasculature on the right side, with one renal artery and vein respectively. On the other hand, there were two renal veins attached to the left IVC (Figure 4). No other obvious kidney anomaly was observed. The left testicular vein drained directly into the left IVC trunk while the right one drained into the inferior left renal vein.

Duplicated IVC has certain implications perioperatively. Knowledge of this vascular anomaly is of essence when performing any surgery involving the retroperitoneum. Vascular injury in vasculature of such calibre can rapidly lead to catastrophic outcomes. More importantly, once duplicated IVC is apparent, surgeons must pay attention to other associated (both vascular and anatomical) anomalies described above. This is of utmost importance in surgery in proximity of the kidneys and in procedures in which ligation of renal vasculature is required. In our patient, the venous anastomosis was in very close proximity to the head of the pancreas. A failure to recognise this anatomical variant may lead to damage to the typically thin-walled, dilated and tortuous vessels and subsequent catastrophic bleeding [10].

The lymphatic system generally follows the vasculature pattern and therefore, lymph node sampling and/or dissection in malignancy may need to be adjusted to account for the unusual patterns of lymph node metastases. Moreover, duplicated IVC and its associated vascular anomalies can often be mistaken as lymphadenopathy, ureteric dilatation, and other intra-abdominal pathology that may potentially lead to unnecessary investigation, over-staging and incorrect decision making [11-12].

Duplicated IVC can also complicate postoperative management. A case series by Tong and Gu highlighted the importance of recognising a duplicated vena cava in the event of deep vein thrombosis in order to successfully place an IVC filter [13]. In our case, our patient commenced on prophylactic anticoagulation shortly after the procedure and did not experience any thrombotic event postoperatively.

## Conclusions

In summary, duplicated IVC is a rare venous anomaly that may be discovered during perioperative imaging and work up. It is important to be recognised, especially procedures involving the retroperitoneum, to minimise the risk of incomplete lymph node dissection and life-threatening bleeding.
